# The impact of the psychodynamic model of depressive symptoms, psychological resilience, and egoism on the well-being among college students: a hybrid three-stage Fuzzy Delphi and structural equation modeling methodology

**DOI:** 10.3389/fpsyg.2025.1603443

**Published:** 2025-08-19

**Authors:** Wang Ting, Peng Qian, Sui Xiaohan

**Affiliations:** Office of the Vice President, Shandong College of Traditional Chinese Medicine, Yantai, Shandong, China

**Keywords:** psychodynamic model, depressive symptoms, psychological resilience, psychological well-being, Fuzzy Delphi method

## Abstract

**Background:**

The rising prevalence of depressive symptoms among college students has raised significant concerns regarding their mental and physical wellbeing. Grounded in psychodynamic theory, this study examines how depressive symptoms, psychological resilience, and egoism collectively influence psychological wellbeing. While existing literature acknowledges these factors independently, their integrated effects remain underexplored. This research addresses this gap by proposing psychological resilience as a mediator and egoism as a moderator, offering a novel theoretical framework for understanding student wellbeing dynamics in higher education contexts.

**Methods:**

The study employed a mixed-methods approach, combining expert validation with large-scale student data. Ten specialists evaluated wellbeing determinants via a Fuzzy Delphi questionnaire. Quantitative analysis involved 1,336 students from a Chinese public university, with data collected through validated scales. Structural equation modeling (SEM) tested hypotheses regarding: (1) depressive symptoms' direct effects, (2) resilience's mediating role, and (3) egoism's moderating influence. The robust methodology enabled simultaneous examination of these interrelated factors while controlling for academic and social stressors.

**Results:**

The findings demonstrate a direct correlation between depressive symptoms and students' well-being, alongside a predominantly positive indirect effect of psychological resilience and egoism on psychological well-being, primarily linked to the advantageous impact of alleviating depressive symptoms among college students. This study's findings demonstrate the detrimental effect of students' depressive symptoms on their well-being in education, implementing psychological resilience and egoism as strategic interruption tools.

**Implications:**

This research provided empirical evidence and thorough statistical analysis demonstrating the simultaneous presence of both positive and negative effects, facilitating the resolution of existing discrepancies in literature. This research introduced a novel instrument for evaluating student well-being, facilitating further investigations inside the university educational context from a fresh research perspective.

## 1 Introduction

The prevalence of depressive symptoms among current students is becoming more concerning, frequently resulting in severe repercussions for their well-being. The relationship between depressive symptoms and students' well-being is a critical area of research, particularly highlighted during the COVID-19 pandemic. Studies indicate that depressive symptoms significantly correlate with various aspects of well-being, including life satisfaction, physical health, and overall psychological health (Ochnik et al., 2024). A moderate positive connection exists between depressive symptoms and psychological well-being in teenagers (Alshehri et al., 2023). Life satisfaction and physical health were recognized as protective factors against depressive symptoms, highlighting the necessity for therapies that improve these dimensions (Ochnik et al., 2024). Variables such as family income and parental occupation were significantly associated with depressive symptoms, indicating that socio-economic status plays a role in students' well-being (Alshehri et al., 2023). While existing research establishes a significant correlation between depressive symptoms and students' well-being – encompassing factors like life satisfaction, physical health, overall psychological health, and socioeconomic status (family income, parental occupation) – there remains a critical gap. Specifically, the relationship between depressive symptoms and well-being understood through the lens of micro psychological activities (such as psychological resilience and egoism) is inadequately explored. This gap limits our understanding of how these fundamental cognitive and emotional processes contribute to, or protect against, depressive symptoms in students, particularly when considered alongside established socioeconomic and broader well-being factors.

This study examined the relationship between depressive symptoms and students' well-being, with psychological resilience serving as a mediator and egoism as a moderator. Psychological resilience has been shown to reduce the impact of depressive symptoms on well-being. For instance, higher resilience levels correlate with improved quality of life in individuals with depression, suggesting that resilience can shield from the adverse effects of depressive symptoms (Novak and Lev-Ari, 2023). Studies indicate that resilience traits, such as hardiness, can lessen the negative correlation between depression and well-being, allowing individuals to maintain a fulfilling life despite depressive symptoms (Chuning et al., 2024). Egoism can mitigate the connection between depression and well-being by affecting emotional regulation and coping mechanisms. Elevated egoism (positive self-concept) and sophisticated defense mechanisms correlate with enhanced well-being and reduced depression (Padmanabhanunni and Pretorius, 2022).

Previous research exclusively utilized SmartPLS to testify the relation between depressive symptom and students' well-being; this study employs a combination of fuzzy Delphi theory and SmartPLS. The integration of FDM and SmartPLS can yield more robust educational models by amalgamating qualitative expert insights with quantitative data analysis (Suib et al., 2024). The integration of FDM and SmartPLS can provide more robust educational models by amalgamating qualitative expert insights with quantitative data analysis. This combination improves the capacity to obtain expert consensus while concurrently examining intricate correlations among educational variables (Suib et al., 2024).

Interventions addressing depressive symptoms across core domains (emotional, cognitive, somatic, behavioral) are consistently associated with enhanced student well-being, supported by a significant positive correlation with psychological well-being (Park et al., 2023). Heightened psychological resilience within specific subdimensions highlights the critical role of structured social support systems in alleviating stress within socially demanding environments (Çaglar et al., 2025). Psychological resilience acts as a vital protective influencing factors, mitigating symptom impact through enhanced emotional regulation and adaptive coping, particularly within demanding academic settings (Çaglar et al., 2025). Furthermore, egoism significantly moderates the link between depressive vulnerability and interpersonal challenges, suggesting a stable self-concept aids in navigating stressors without succumbing to depression (Han and Lee, 2019). Seo (2022) confirms egoism's protective function through its alleviation of school stress and depression. Therefore, this study aims to examine the interrelationships between core depressive dimensions (emotion, cognition, somatic, behavioral), psychological resilience, and egoism in shaping college students' mental health and well-being.

Specifically, based on this empirical foundation and the proposed research purpose, we propose the following hypotheses:

**Hypothesis 1:** There is a significant relationship between depressive symptoms and students' well-being.
**Hypothesis 2:** There is a significant relationship between depressive symptoms (emotion perception) and students' well-being with psychological resilience as a mediator.
**Hypothesis 3:** There is a significant relationship between depressive symptoms (cognition perception) and students' well-being with psychological resilience as a mediator.
**Hypothesis 4:** There is a significant relationship between depressive symptoms (somatic perception) and students' well-being with psychological resilience as a mediator.
**Hypothesis 5:** There is a significant relationship between depressive symptoms (behavioral activity) and students' well-being with psychological resilience as a mediator.
**Hypothesis 6:** There is a significant relationship between depressive symptoms and students' well-being with egoism as a moderator.

In conclusion, this research significantly advances our understanding by moving beyond a simple link between depression and student well-being. It illuminates the crucial mediating pathway of psychological resilience across specific depressive dimensions and identifies egoism as a key moderator. These insights are vital for developing targeted, mechanism-driven, and personalized interventions to effectively promote resilience and enhance well-being in diverse student populations facing depressive challenges. This framework delivers actionable pathways for educational institutions to translate theoretical insights into structured support systems, directly addressing the multifaceted nature of student depressive syndrome through evidence-based academic practice.

## 2 Literature review

### 2.1 Psychodynamic model of depressive symptoms

**Figure 1:**
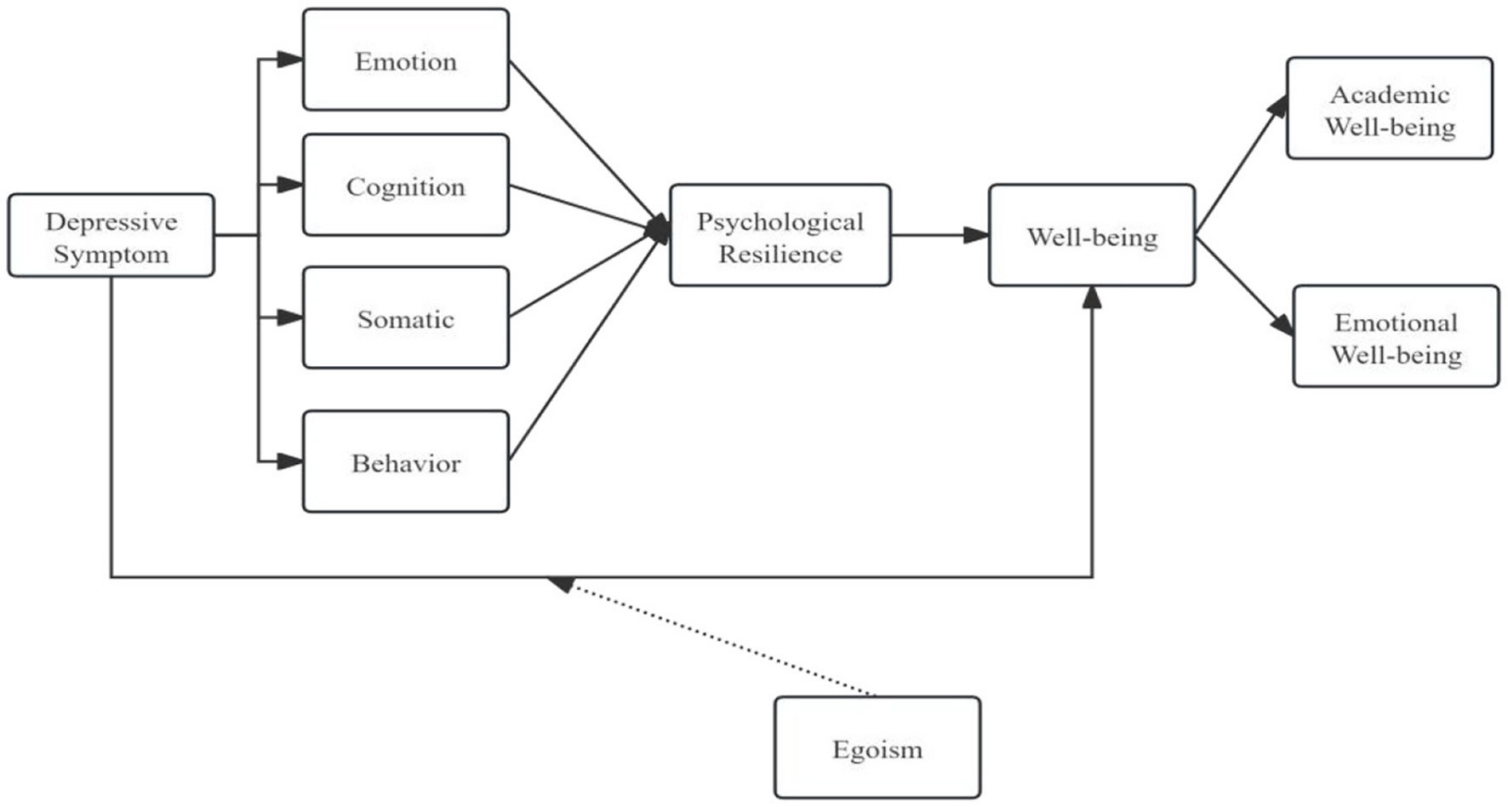
Conceptual framework.

The psychodynamic model of depressive symptoms included emotional, cognitive, physical, and behavioral aspects that collectively improve students' well-being. Emotional reactions, including wrath and disappointment, are fundamental to the psychodynamic interpretation of depression. These sentiments frequently arise from narcissistic hurts and processes of idealization (Busch, 2021). Cognitive patterns, such as negative self-perceptions and maladaptive attitudes regarding relationships, might sustain depressive symptoms and obstruct well-being (Schauenburg, 2020). Somatic symptoms frequently present as physical ailments associated with emotional turmoil. Utilizing psychodynamic therapy to address these issues helps enhance emotional regulation (Joyce-Beaulieu and Zaboski, 2020). Behavioral components pertain to the influence of depression symptoms on social relationships and academic achievement. Therapeutic therapies seek to alter these habits, promoting enhanced social interaction and academic achievement (Joyce-Beaulieu and Zaboski, 2020). The conceptual structure of this investigation is depicted in Figure 1. Consequently, an assessment of the hypothesis concerning the impact of the Psychodynamic Model was feasible.

Research indicates that depressive symptoms significantly influence students' overall well-being, affecting their academic engagement and psychological health (Zhang et al., 2024). Some studies indicate depressive symptoms, though prevalent among students, do not inevitably impair well-being when support systems and adaptive strategies exist—reflecting a nuanced relationship requiring further investigation (Zhang et al., 2024).

### 2.2 The mediating effect of psychological resilience on the relationship between depressive symptoms and students' well-being

The correlation between depressive symptoms and students' well-being is substantially affected by psychological resilience, which serves as a mediating variable. Studies demonstrate that resilience not only alleviates the effects of stress and depression symptoms but also improves general well-being in students.

Psychological resilience acts as a mediator against depressive symptoms, enhancing mental health outcomes (Yan et al., 2024). Research indicates that resilience mediates the association between multiple characteristics, including perceived social support and coping mechanisms, and depression (Dong et al., 2024). This indicates that increased resilience may result in less depression symptoms and enhanced well-being.

Somatic manifestations of depression, including fatigue and sleep difficulties, may be mitigated with resilience-enhancing therapies, resulting in enhanced well-being (Cengiz et al., 2024). Behavioral resilience fosters proactive coping mechanisms, diminishing the probability of depressive episodes and enhancing participation in constructive activities (Dong et al., 2024). According to Gilchrist et al. (2023), emotional resilience enables students to preserve a positive perspective in the face of depressive symptoms, which can contribute to their overall well-being. Cognitive resilience is characterized by adaptive thinking patterns that reduce the influence of negative emotions, thereby promoting a more positive mental state (Yu and Zhou, 2024).

Research-based evidence confirms psychological resilience functions as a critical protective mechanism, mitigating depressive symptoms' adverse impact on well-being through enhanced emotional regulation and adaptive environmental coping strategies. This mediating role proves particularly vital in academic contexts, where pervasive stressors threaten students' mental health and psychological well-being.

### 2.3 The moderating effect of egoism on the relationship between depressive symptoms and students' well-being

Egoism represents an adaptive, structurally stable and internalized sense of self-worth and identity, enabling genuine autonomy and relatedness without dependency on external validation (Tilley, 2022). This stands in sharp contrast to narcissism—a maladaptive constellation characterized by fragile self-esteem, a pervasive need for admiration, entitlement, exploitativeness, and lack of empathy, which stems from underlying vulnerability and defensively manifests as grandiosity (Fadhila, 2024). While both egoism and ego-resilience are adaptive ego capacities, they function distinctly: Egoism establishes the foundational “solidity” of the enduring self-concept, whereas ego-resilience reflects the dynamic capacity to flexibly modulate cognitive, emotional, and behavioral responses to situational demands (Han and Lee, 2019). Crucially, the structural stability afforded by egoism provides the necessary platform upon which the dynamic adaptability of ego-resilience operates.

Egoism, functioning as a moderating factor, can significantly influence the relationship between depressive symptoms and student well-being (Besharat et al., 2017). Defined by a stable, coherent sense of self rather than self-centeredness, egoism may enhance well-being by providing a foundational protective factors against depressive symptoms, enabling students to maintain a more consistent sense of identity and worth amidst challenges (Assor et al., 2021).

Egoism moderates depressive vulnerability's impact on interpersonal dysfunction among university students. Elevated egoism levels correlate with enhanced interpersonal problem-solving capacities, thereby reducing depression susceptibility (Han and Lee, 2019).

## 3 Fuzzy Delphi data analysis

The Delphi approach, including the Delphi procedure and fuzzy theory, was employed to examine the perspectives of professionals knowledgeable in this field of study. This approach has been employed to attain anonymity, iteration, regulated feedback, and statistical group response. The implementation of the fuzzy Delphi technique necessitates a panel of experts including between nine and fifteen individuals (Daud et al., 2024). Consequently, this study focused on 10 experts with at least 5 years' expertise. This arises from the divergence of perspectives on this subject. For this study, 10 experts from China's vocational education were recruited. Table 1 displayed the particular circumstances of the experts. These 29 variables influencing student well-being were derived from a 2003–2023 meta-analysis (Xiong et al., 2025). They were structured into an expert's scale to collect expert opinions. Ten experts evaluated each variable using this instrument. The opinions of these professionals were solicited, as they had a responsibility to provide guidance about the improvement of college students' well-being. The questions utilized a 5-point Likert scale, with the five levels designated as “completely disagree,” “disagree,” “neutral,” “agree,” and “completely agree,” assigned corresponding reverse integers from 1 to 5. This method has six steps, beginning with experts assessing the significance of the variable, followed by the conversion of linguistic variables into fuzzy numbers, and culminating in the use of the vertex method to calculate the distance between the average values.


(1) $$\begin{array}{l} d\left(\tilde{m},\tilde{n}\right)=\sqrt{\frac{1}{3}\left[{{\left(m_{1}-n_{1}\right)}^{2}+{\left(m_{2}-n_{2}\right)}^{2}+\left(m_{3}-n_{3}\right)}^{2}\right]} \end{array}$$

**Table 1:** Experts with specific qualities.

**Age group**	**Specific position**	**Years of experience**
31–40	Instructor	6–10
21–30	Instructor	6–10
21–30	Director	11–15
21–30	Psychological teacher	16–20
41–50	Principal	11–15
21–30	Division chief	6–10
41–50	Teaching staff	6–10
21–30	Psychological teacher	1–5
31–40	Teaching staff	6–10
41–50	Section chief	11–15

Table 1 delineated the characteristics of the experts and their replies to questions concerning the factors that affected students' well-being. The ages of the experts, as shown in Table 1, ranged from 21 to 50 years. They occupied various roles, including section chief, division chief, faculty member, director, principal, and instructor. The professionals' expertise ranged from 5–20 years. This study sought to determine the views of seasoned administrative personnel and proficient educators regarding the elements that impacted students' well-being. To ensure robust dimension validation grounded in both scholarly rigor and practical relevance, experts were purposively selected based on three integrated criteria: (a) a minimum 5 years' specialized experience in clinical psychology, educational psychology, or adolescent mental health intervention; (b) demonstrated scholarly contributions through publications in depression, resilience, or ego psychology; and (c) active engagement in university counseling or student well-being programming. This triangulation of credentials ensured panelists possessed the necessary theoretical-practical nexus to effectively evaluate student well-being dimensions—particularly micro-psychological constructs like egoism—within authentic educational ecosystems (Stern et al., 2020).

In the fourth phase, according to Khaira and Ibrahim (2024), if the disparity between the average and the expert evaluations is below the threshold of 0.2, all experts are deemed to have reached an agreement.

If the proportion of reaching group consensus among the m x n ratings of options and n criteria weights exceeds 75% (Khaira and Ibrahim, 2024), proceed to step 5; otherwise, a second round of survey is necessary.

The fifth step encompasses imprecise evaluations:


(2) $$\begin{array}{l} \tilde{A}=\\ \;\left[\begin{array}{c} {\tilde{A}}_{1}\\ \vdots \\ {\tilde{A}}_{m} \end{array}\right]where\;{\tilde{A}}_{i}={\tilde{r}}_{i1}⊗{\tilde{W}}_{1}⊕{\tilde{r}}_{i2}⊗{\tilde{W}}_{2}⊕\ldots ⊕{\tilde{r}}_{in}⊗\\ \;{\tilde{W}}_{n}\;\left(i=1,\ldots ,m\right) \end{array}$$

The six-step process for each of the available alternatives, the fuzzy evaluation Ã_*i*_ = (*a*_*i*1_, *a*_*i*2_, *a*_*i*3_) was defuzzified by


(3) $$\begin{array}{l} a_{i}=\frac{1}{4}\left(a_{i1}+{2a}_{i2}+a_{i3}\right) \end{array}$$

**Figure 2:**
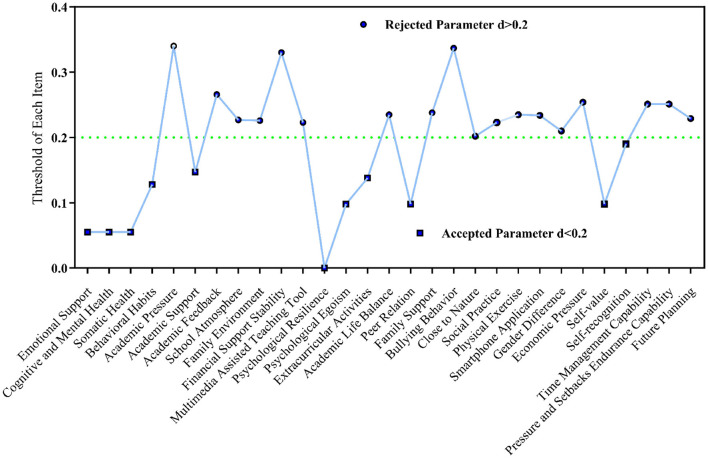
Accepted and rejected parameters according to a threshold value (d).

The fuzzy Delphi Method aimed to achieve expert consensus with a questionnaire utilizing the Likert scale. A fuzzy Delphi method was employed to transform the results into fuzzy integers. The language variables were converted into fuzzy scales. The mean group consensus was 80%, rendering this version acceptable. Consensus would be achieved if the average group agreement exceeded 75%. If the percentage is below 75%, a new poll must be administered due to the absence of consensus (Buryanov et al., 2023). The principal acceptable and rejected dimensions, as determined by fuzzy findings, were illustrated by the number below (Figure 2).

Figure 2 indicated that the d values for emotional support, cognitive and mental health, somatic health, behavioral habits, extracurricular activities, and academic support were below 0.2. The six components pertained to the dimension of depressive symptoms. The values of psychological egoism, self-worth, and self-recognition that constituted the egoism dimension were below 0.2 (Peng et al., 2024). The value of psychological resilience was below the threshold of 0.2. Ultimately, three dimensions—depressive symptoms, psychological resilience, and egoism—were identified as the primary determinants impacting the enhancement of students' well-being.

## 4 Research design

This study selected one public institutions in Shandong Province that offered Traditional Chinese Medicine (TCM) programs as the research subjects. A mixed-methods strategy, integrating Fuzzy Delphi method and SmartPLS, was adopted. The sequential mixed-methods design was theoretically anchored in complexity theory, where qualitative expert consensus (Phase 1) captured context-bound conceptualizations of dynamic mental health constructs (depressive symptoms, egoism, resilience), enabling subsequent quantitative modeling (Phase 2) to test generalized relationships while preserving ecological validity (Stern et al., 2020). The quantitative phase involves modeling the relationships identified in the qualitative phase to test their generalizability across larger populations. This phase uses statistical methods to validate the findings and explore the broader applicability of the insights gained (Wackerhagen et al., 2022). Initially, structured expert interviews were performed to examine the elements affecting the students' well-being. The expert questionnaire was designed based on the research objective and the previous literature review, encompassing 29 items. After the analysis of expert questionnaire, three influencing factors related to students' well-being were decided such as depressive symptoms, egoism and psychological resilience. The “Multidimensional Assessment Scale of Mental Health for College Students” was developed based on the findings from the expert questionnaire analysis. The scale comprised four dimensions. The perspective on depression symptoms encompassed four aspects and 20 items derived from the UPI scale. The University Personality Inventory demonstrated exceptional internal consistency, achieving an overall alpha of 0.97 and individual variable alphas ranging from 0.83 to 0.91, indicating its dependability as a mental health screening tool (Sugawara et al., 2020). Five items pertaining to psychological resilience were derived from the psychological resilience scale. Psychological resilience scales are essential tools for evaluating an individual's ability to adapt and thrive during adversity (Reinwarth et al., 2024). Five items were selected from the psychological egoism scale to assess the concept of egoism. Psychological egoism scales are tools designed to evaluate the extent to which individuals prioritize self-interest in their thoughts and behaviors (Overall and Gedeon, 2022). Ten indicators of student well-being were derived from the school well-being profile (Monroy et al., 2024). The well-being dimension was divided into two components: academic well-being and emotional well-being. The “Multidimensional Assessment Scale of Mental Health for College Students” totally included 40 items.

This research was carried out in higher education institutions in China. Consequently, the research was conducted in the first quarter of 2025. Ten experts of this study were selected from the target population by purposive sampling procedures. Purposive sampling aims to select individuals possessing particular features or experiences pertinent to the study inquiry (Magnone and Yezierski, 2024). For the second phase (quantitative methodology), simple random sampling methodology was adopted. For quantitative part, simple random sampling methodology was adopted. In SRS, each unit in the population has an equal probability of being selected. This can be achieved by drawing units one by one, either with or without replacement, from the population (West, 2016). A total of 1382 students participated in this research, with the majority being female and the remainder male. All participants were aged 18 and above, excluding minors. In total, 1,336 students completed the questionnaires, resulting in an 97% response rate, which met the criteria of cross-sectional studies. Researchers intending to employ Structural Equation Modeling (SEM) must ensure a minimum of 10 participants per estimated parameter. Given 40 parameters, this necessitates at least 400 subjects (Shinya and Takiyama, 2024). Consequently, the sample size exceeding 400 cases was adequate for deriving significant results.

## 5 Research procedure

This research endeavor commenced with the assessment and endorsement of the Human Research Ethics Committee. Subsequently, the pertinent documents were gathered and examined to construct an expert questionnaire. The questionnaire was dispatched to 10 experts via email to elicit their responses regarding the significant aspects impacting students' well-being. Multidimensional Assessment Scale of Mental Health for College Students was developed and finalized based on expert analysis results. Upon the completion of the initial edition of the scale, a pilot study was executed utilizing the instrument with a sample from various majors of students, facilitating the refinement of the questionnaire for its final iteration. The students were subsequently invited to participate in this research voluntarily. The participants received the informed consent statement, which outlined the research objectives, duration, instrument instructions, data confidentiality assurances, and expected outcomes. Participants who executed this informed consent form consented to engage in the study on-site by completing the questionnaire. Individuals unable to attend in person answered the survey through an online link.

**Figure 3:**
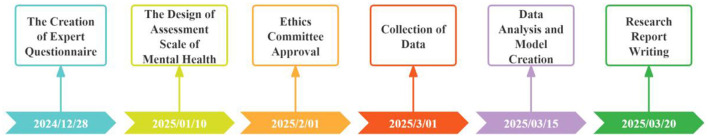
Research procedure.

The rights of the subjects were consistently upheld throughout the investigation. Participants were informed that they could withdraw at any moment. The participants' physical and mental well-being, data confidentiality, the right to anonymity, and other ethical requirements pertaining to human subjects were safeguarded. Figure 3 illustrated the progression of the study.

## 6 Data analysis and model validation

The Partial Least Squares (PLS) modeling was employed to assess the study framework utilizing SmartPLS v4.0, as it analyzed data independent of sample size and normality. Moreover, SmartPLS possesses enhanced capabilities for predicting and analyzing causal linkages (Lin and Huynh, 2024). Discriminant and convergent validity were assessed prior to evaluating the study model hypotheses. Subsequently, the details were categorized by metrics representing the primary variables and their dimensions for the dependent variable (De La Rubia, 2019). The criteria for assessing closer suitability included factor loading exceeding 0.7; Average Extracted Variance (AVE) more than 0.4; and Cronbach's Alpha (CA) and Combined Reliability (CR) of internal consistency reliability surpassing 0.7 (De La Rubia, 2019).

### 6.1 Convergent and discriminant validity

The measurement models were evaluated for construct validity and reliability, encompassing both convergent and discriminant validity. Cronbach's alpha coefficients were evaluated for all core variables in the measuring framework of this study to determine construct reliability. This research determined that each Cronbach's alpha coefficients varied from 0.838 to 0.906, exceeding the proposed threshold of 0.7 (Hayat, 2024). The composite reliability (CR) factors varied from 0.884 to 0.919, surpassing the threshold of 0.7 (Lai, 2020). Average Variance Extracted (AVE) values were employed to assess convergent validity. All AVE values varied between 0.566 and 0.807, above the suggested minimum of 0.50 (Da Costa et al., 2022). The following Table 2 showed that construct reliability was satisfactory, since Cronbach's alpha coefficients, composite reliability and AVE values met the criteria requirements.

**Table 2:** Evaluation of validity and reliability value.

**Dimensions**	**Items**	**Outer loadings (>0.7)**	**Cronbach's alpha (>0.7)**	**Composite reliability (>0.7)**	**Composite reliability (>0.7)**	**Average variance extracted (AVE) (>0.5)**
Depressive symptom	So1	0.791	0.908	0.909	0.929	0.685
	So2	0.851				
	So3	0.831				
	So4	0.843				
	So5	0.8				
	So6	0.85				
	Be1	0.759	0.825	0.83	0.884	0.656
	Be2	0.805				
	Be3	0.867				
	Be4	0.806				
	Co1	0.812	0.916	0.917	0.937	0.748
	Co2	0.862				
	Co3	0.892				
	Co4	0.875				
	Co5	0.882				
	Em1	0.859	0.919	0.919	0.939	0.755
	Em2	0.853				
	Em3	0.889				
	Em4	0.864				
	Em5	0.877				
Egoism	Eg1	0.837	0.918	0.926	0.938	0.752
	Eg2	0.886				
	Eg3	0.879				
	Eg4	0.886				
	Eg5	0.846				
Emotional well-being	EW1	0.913	0.919	0.92	0.943	0.806
	EW2	0.9				
	EW3	0.922				
	EW4	0.854				
Academic well-being	AW1	0.811	0.903	0.907	0.928	0.722
	AW2	0.884				
	AW3	0.903				
	AW4	0.87				
	AW5	0.772				
Psychological resilience	PR1	0.905	0.96	0.961	0.968	0.835
	PR2	0.937				
	PR3	0.888				
	PR4	0.931				
	PR5	0.92				

So, Somatic; Be, Behavior; Co, Cognition; Em, Emotion; Eg, Egoism; EW, Emotional Well-being; AW, Academic Well-being; PR, Psychological Resilience.

Factor loading is an essential statistical metric employed to evaluate the reliability of indicators across several study situations (Christensen et al., 2025). It measures the correlation between observed variables and underlying latent components, therefore offering insights into the validity of the investigated indicators (Christensen et al., 2025). This approach is especially beneficial in domains like healthcare and psychology, as it enhances measurement instruments and augments data interpretation. Based on Table 2, it demonstrated that all items exhibited factor loadings exceeding the recommended level of 0.7. It indicated that both individuals showed a significant proficiency in clarifying their respective subjects.

The Fornell-Larcker criterion is a well-established approach for evaluating discriminant validity in studies with latent variables. It assesses the distinctiveness of constructs by comparing the square root of the average variance extracted (AVE) for each construct against the correlations among constructs. A construct possesses sufficient discriminant validity if the square root of its Average Variance Extracted (AVE) exceeds its correlations with other constructs (Kamarudin et al., 2023). The Table 3 three illustrated that the mean square root of each dimension surpassed its correlation coefficient with other dimensions, indicating that the data demonstrated differential validity.

**Table 3:** Discriminant validation using Fornell-Lacker method.

**Items**	**AW**	**Be**	**Co**	**Eg**	**Em**	**EW**	**PR**	**So**
AW	0.85							
Be	0.453	0.81						
Co	0.562	0.706	0.865					
Eg	0.506	0.249	0.269	0.867				
Em	0.537	0.687	0.848	0.241	0.869			
EW	0.807	0.51	0.634	0.437	0.609	0.898		
PR	0.737	0.555	0.665	0.438	0.622	0.766	0.914	
So	0.455	0.763	0.731	0.263	0.686	0.528	0.553	0.828

So, Somatic; Be, Behavior; Co, Cognition; Em, Emotion; Eg, Egoism; EW, Emotional Well-being; AW, Academic Well-being; PR, Psychological Resilience.

### 6.2 Significance assessment of the structural model

**Figure 4:**
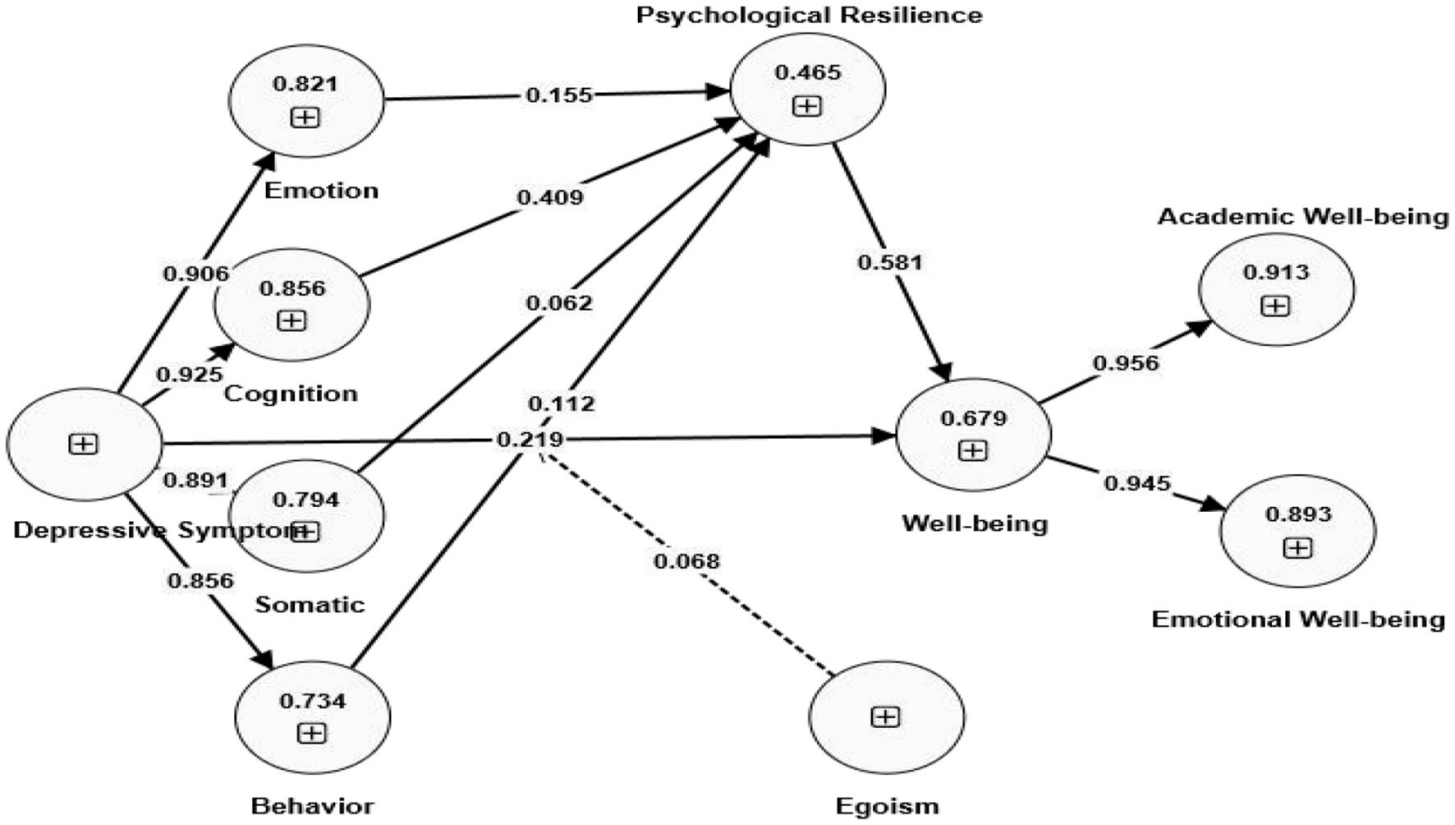
Structural model.

This part aims to delineate the response levels of the study sample persons and analyze them through the arithmetic mean and standard deviation concerning the explanatory axis of depressive symptom and well-being, as seen in Figure 4.

### 6.3 Assessment of the SEM significance

The structural model delineates causal relationships among diverse constructs (independent, moderating, control, mediating and dependent variables) to evaluate the proposed hypotheses of the investigation. Hair et al. (2021) noted that path analysis (analyzed using a one-tailed test), which includes the beta coefficient, standard error, *t*-value, *p*-value and Q^2^ constitutes essential criteria for evaluating the structural model in PLS.

This research employed six hypotheses to address the specified research issues. Parameter estimates for statistical significance and coefficient values were assessed for hypothesis testing using a bootstrapping method grounded in PLS-SEM (Kostanek et al., 2024). The bootstrapping approach, employing 5,000 two-tailed resamples at a significance level of 0.05 with 345 case data, was utilized to assess the significance of path coefficients through bias-corrected confidence intervals (Özkale and Altuner, 2022). The *t*-value for the two-tailed test must be at least 1.96 at a 5% significant level (Roodman et al., 2019).

Table 4 summarized the statistical analysis results for all tested hypotheses. Hypothesis 1 substantiated the correlation between depressed symptoms and students' well-being, as the t-value exceeded 1.96, the *p*-value was < 0.005, and the path coefficient was >0. Consequently, Hypothesis 1 was affirmed, indicating that depression symptoms were diminished, resulting in enhanced student well-being. H2 was supported since the relation between emotion and psychological resilience and the correlation between psychological resilience and well-being was testified since the *t*-value exceeded 1.96, the *p*-value was < 0.005, and the path coefficient was >0. Hypothesis 2 was substantiated as the association between emotion and psychological resilience, as well as the link between psychological resilience and well-being, was validated by a *t*-value exceeding 1.96, a *p*-value below 0.005, and a path coefficient more than 0. The hypothesis H3, which posited that psychological resilience mediated the relationship between cognition and well-being, was supported, as the *t*-value exceeded 1.96, the *p*-value was below 0.05, and the path coefficient was positive. The H4 hypothesis was unsupported, as the *p*-value exceeded 0.05, indicating no correlation between somatic reactions and students' well-being. H5 demonstrated that psychological resilience mediated the relationship between behavior and well-being, substantiating the connections among these variables. H6 demonstrated the positive moderating effect of egoism on the relationship between depressive symptoms and student well-being (*p*-value was 0.00, *t*-value was 4.320, and the path coefficient was 0.068). Though statistically robust, the small coefficient implies only marginal buffering against depression's well-being impacts, aligning with prior findings that self-enhancement motives offer limited protection (Sarzhanova and Nurgabdeshov, 2025).

**Table 4:** Hypotheses tests of study.

**Hypothesis**	**Hypotheses**	**T statistics**	***P*-values**	**Path coefficients**	**Results**
H1	Depressive symptom -> well-being	8.581	0.000	0.219	Supported
H2	Emotion -> psychological resilience	3.898	0.000	0.155	Supported
	Psychological resilience-> well-being	19.481	0.000	0.581	
H3	Cognition -> psychological resilience	9.274	0.000	0.409	Supported
	Psychological Resilience-> well-being	19.481	0.000	0.581	
H4	Somatic -> psychological resilience	1.721	0.085	0.062	Unsupported
	Psychological resilience-> well-being	19.481	0.000	0.581	
H5	Behavior -> psychological resilience	2.988	0.003	0.112	Supported
	Psychological resilience -> well-being	19.481	0.000	0.581	
H6	Egoism -> depressive symptom -> well-being	4.320	0.000	0.068	Supported

### 6.4 Predictive relevance (Q^2^)

Predictive Relevance (Q^2^) assesses the predictive significance of the inner model. This measure is predicated on a sampling technique known as Blindfolding. This method involves omitting a portion of the data matrix, estimating the model parameters, and forecasting the omitted segment (Czasonis et al., 2023).

**Figure 5:**
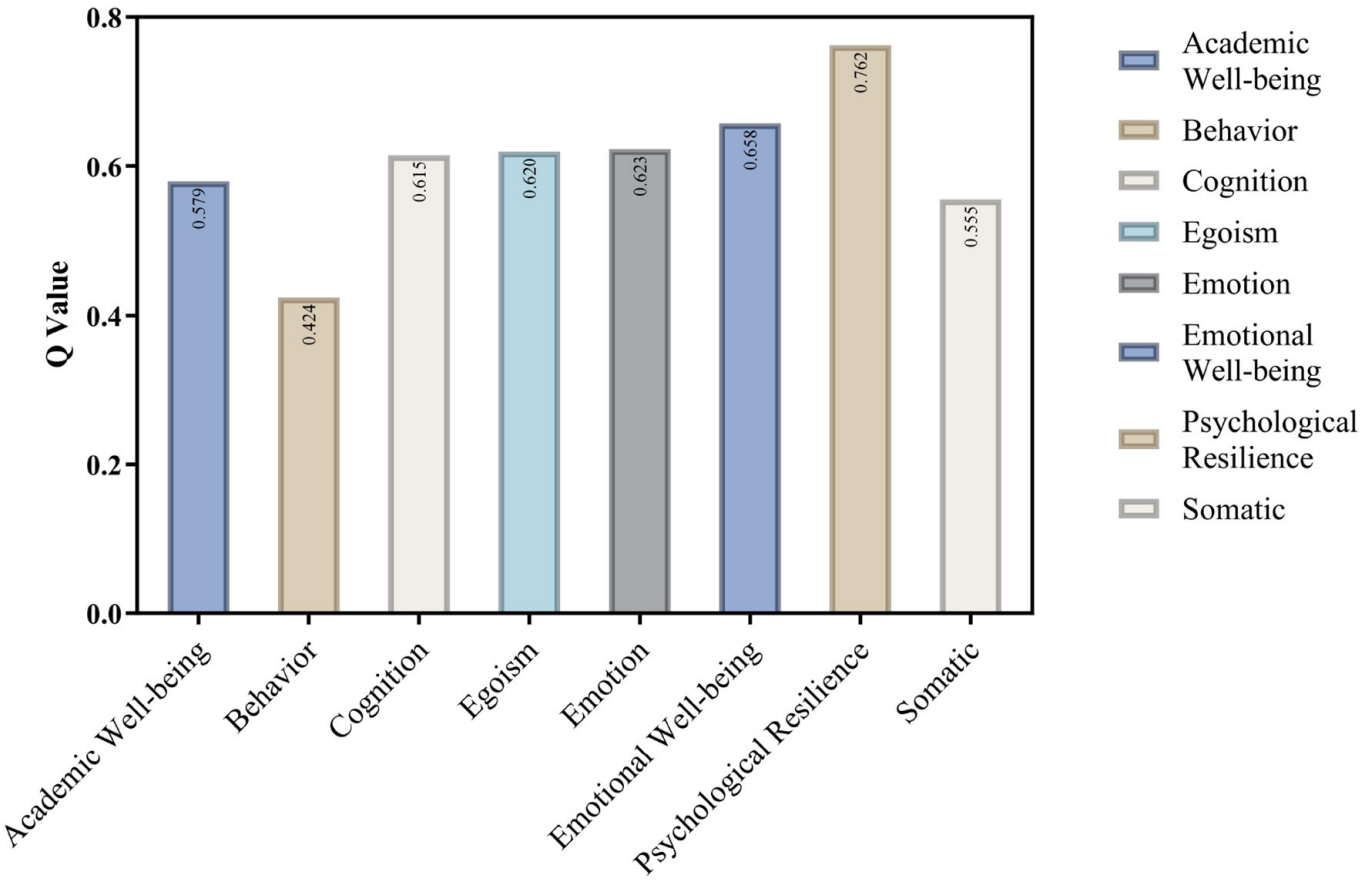
Predictive relevance (Q^2^).

If the Q^2^ result value for a specific endogenous construct exceeds zero, it signifies the predictive importance of the route model for that construct. The Q^2^ values for this study ranged from 0.424 to 0.762, all significantly above zero as illustrated in Figure 5. This demonstrated the efficacy of a model in predicting data from excluded cases, as evidenced by the heightened predictive value of the research variables about the impact of depressive symptoms on students' well-being.

## 7 Discussion of findings

The research objectives and the corresponding methodologies employed to attain them were detailed in Table 5. The initial objective was accomplished by Fuzzy Delphi Analysis, yielding 11 essential influencing factors belonging to 3 dimensions. The second objective was accomplished by SmartPLS analysis, yielding five acceptable hypotheses, as illustrated in Table 6.

**Table 5:** Research objectives and findings summary.

**Objective**	**Method**	**Results**
To identify the influencing factors related to students' well-being based on the fuzzy Delphi Analysis.	fuzzy Delphi method	Eleven influencing factors concerning the students' well-being.
To investigate the impact and importance of depressive symptom on students' well-being with psychological resilience as a mediator and egoism as a moderator.	SmartPLS analysis	The most influencing factors on students' well-being are psychological resilience, depressive symptom, emotion, cognition and behavior.

**Table 6:** Summary of accepted hypotheses.

**Order**	**Hypotheses**	**Results**
H1	There is a significant relationship between depressive symptoms and students' well-being.	Supported
H2	There is a significant relationship between depressive symptoms (emotion perception) and students' well-being with psychological resilience as a mediator.	Supported
H3	There is a significant relationship between depressive symptoms (cognition perception) and students' well-being with psychological resilience as a mediator.	Supported
H5	There is a significant relationship between depressive symptoms (behavioral activity) and students' well-being with psychological resilience as a mediator.	Supported
H6	There is a significant relationship between depressive symptoms and students' well-being with egoism as a moderator.	Supported

Table 6 indicated that hypotheses H1, H2, H3, H5, and H6 were accepted, whereas hypothesis H4 was rejected. Nonetheless, the somatic situation was dismissed, which did not imply that it lacked an effect on the pupils' well-being. It suggested that somatic conditions remained a significant influencing factor for students' well-being, according to the opinions of experts. While Hypothesis 4 (predicting a significant relationship between somatic symptoms and egoism) was statistically rejected in our quantitative analysis, the persistent emphasis on somatic manifestations by clinical experts during the Delphi phase warrants methodological reflection. This divergence may stem from the following two interrelated factors: First, measurement granularity—experts contextualized somatic symptoms as embodied expressions of psychological distress (e.g., fatigue as intertwined with cognitive depletion), whereas our Likert-scale instrument may have captured these phenomena reductively, obscuring clinically meaningful nuances (Shen et al., 2022). Second, respondent awareness—students experiencing subsyndromal depression often lack somatic symptom attribution to mental health struggles, leading to underreporting in self-report measures (McGhie-Fraser et al., 2024). In comparison to the impact of somatic conditions, depressive symptoms, emotions, cognition, behavioral activity, and egoism were deemed more significant. These contributing elements must be given greater significance to foster students' mental resilience, mitigate depressive symptoms, and increase overall well-being.

Depressive syndrome impacts student well-being across emotional, cognitive, somatic, and behavioral dimensions (Park et al., 2023). College-based interventions show promise in addressing this relationship: resilience training fosters emotional growth (Cengiz et al., 2024), while positive psychology courses effectively teach happiness and resilience skills (Yang, 2023). A key protective factor, egoism - characterized by emotional regulation and curiosity - significantly enhances well-being by promoting autonomy, enjoyment, and stronger social connections (Leow et al., 2024). These findings highlight the importance of developing psychological resources to counter depressive symptoms while building student well-being (Park et al., 2023). Notably, while cognitive, emotional, and behavioral factors show limited direct impact on depression severity, they remain fundamental components of overall psychological well-being.

Depressive symptoms among Chinese college students, exacerbated by academic and socioeconomic pressures, necessitate enhanced mental health interventions. Institutions must prioritize psychological resilience and adaptive self-interest as key protective factors. By integrating these elements into curricula, campus policies, and extracurricular programs, universities can systematically strengthen students' coping capacities. The systematic implementation of complementary protective measures (resilience training and healthy ego development) would serve to both strengthen students' self-cognition and support enduring psychological well-being. This proactive approach addresses both immediate mental health needs and long-term adaptive functioning, aligning student support with China's evolving educational demands.

## 8 Implications of the study

### 8.1 Theoretical implications

This study enhances the psychodynamic framework by incorporating depressive symptoms, psychological resilience, and egoism to elucidate psychological well-being in college students. The results corroborate the psychodynamic perspective by illustrating the interaction between internal tensions (depressive symptoms), ego defensive mechanisms (egoism), and adaptive coping (resilience) in affecting well-being. The study validates resilience as a mediator, aligning with positive psychology and highlighting that adaptive traits can reduce mental health risks. Furthermore, the incorporation of egoism as a moderator enhances psychodynamic theory, demonstrating how self-centered inclinations may mitigate or intensify depressed outcomes.

This study adopts Block and Block's (1980) psychoanalytic definition of egoism (as ego strength)—the structural capacity to maintain a stable self-identity—distinct from colloquial self-centeredness. This reconceptualization is justified by three evidence-based functions: (1) It enables authentic relatedness through well-defined ego boundaries (Tilley, 2022); (2) It protects from depressive fragmentation during stress (Fadhila, 2024); and (3) It provides the stable “I” necessary for resilient “we” functioning (Li, 2023). Thus, operationalizing egoism as promotable reflects its empirically validated role in well-being.

The Fuzzy Delphi method's expert agreement improves theoretical precision, integrating psychodynamic paradigm while providing a systematic framework for well-being determinants. The SEM analysis offers empirical validation for the dynamic interactions among these dimensions, addressing differences in the literature by affirming both protective (resilience) and ambivalent (egoism) aspects.

This research also enhances methodological discourse by introducing an innovative well-being evaluation instrument, prompting future investigations to embrace multidimensional frameworks. This contextualization of findings within China's educational framework emphasizes cultural subtleties in egoism and resilience, advocating further cross-cultural psychodynamic investigations. The study emphasizes the necessity for integrative models that amalgamate intrapsychic (psychodynamic) and interpersonal (resilience) aspects in mental health research.

### 8.2 Practical implications

To improve student well-being and alleviate depressive symptoms, institutions should prioritize programs that cultivate psychological resilience and adaptive egoism.

To successfully mitigate increasing depressed symptoms among college students, institutions must establish comprehensive resilience-building programs and encourage adaptive egoism. Universities can improve students' coping abilities and mental health by including cognitive-behavioral seminars, mindfulness training, peer support networks, and resilience-oriented counseling. Concurrently, promoting healthy self-focus through autonomy-supportive courses and conversations on ethical egoism enables students to reconcile self-care with social involvement. This dual strategy provides students with essential psychological resources to alleviate students' desperation and maintain enduring well-being, fostering a more supportive academic atmosphere.

Educate educators to identify depressed symptoms and direct students to mental health options. Establish early screening initiatives to identify at-risk students and deliver prompt solutions. Cultivate a campus environment that encourages help-seeking behavior and self-advocacy.

By integrating resilience training with systematic egoism development, colleges can furnish students with competencies to mitigate depression while preserving psychological well-being. This dual strategy enables students to manage academic stress efficiently, utilizing the strength of self-identity as a protective barrier against mental health issues.

## 9 Limitations and future directions of the study

This research employed a mixed methodology via a survey for experts' feedbacks and a survey for students' data collection. The questionnaire design incorporated various characteristics from prior work while excluding factors such as emotional quotient, blunt capability, adversity quotient, and emotional support. In the future, additional factors should be considered to enhance the theoretical framework and construct a more robust architecture with clearly defined hierarchical dimensions. Owe to time limitation, the study employed the cross-section approach. Consequently, the transversal nature of the data collection approach obstructs the accurate evaluation of students' mental development. Consequently, the longitudinal technique is suitable as it can evaluate the robustness of the correlations.

In considering the study's limitations, future research should investigate associated variables to enhance the theoretical framework. A comparative analysis between two distinct sectors will elucidate the phenomena more comprehensively. Future research may adapt the proposed model in response to emerging scenarios to enhance its theoretical and practical implications. The forthcoming study should employ a longitudinal methodology to yield more comprehensive and dynamic data that cross-sectional studies have failed to provide, so enhancing the understanding of long-term trends and causal relationships.

## 10 Conclusion

Psychological resilience plays a crucial and beneficial role in relation to depressive symptoms and student well-being. To alleviate students' depressive symptoms and enhance students' well-being, the college should emphasize the development of psychological resilience through diverse interventions, including: (1) conducting cognitive-behavioral workshops to improve coping strategies, (2) incorporating mindfulness-based stress reduction (MBSR) programs into the curriculum, (3) creating peer support networks to enhance social connectedness, and (4) offering accessible counseling services featuring resilience-oriented therapy modules. The factor of egoism acts as a crucial protective moderator, significantly mitigating the adverse effects of depressive symptoms on students' psychological well-being. Thus, the college should prioritize the cultivation of students' egoism to protect against students' depressive feelings and obtain students' sense of well-being. Colleges can effectively equip students with self-preservation skills that protect against depressive tendencies by implementing structured programs (e.g., autonomy-supportive curricula, resilience-focused cognitive-behavioral training workshops, and ethical egoism discussions). This multifaceted approach enables students to manage academic pressures while preserving well-being, framing egoism not as narcissism but as a robust aspect of self-identity—a vital moderator in the link between depression and well-being.

## Data Availability

The original contributions presented in the study are included in the article/supplementary material, further inquiries can be directed to the corresponding author.
